# Acknowledgement of reviewers for 2023

**DOI:** 10.1590/2175-8239-JBN-2024-P001en

**Published:** 2024-02-23

The Editor-in-Chief, the Deputy Editor, and the Associate Editors of the Brazilian Journal of Nephrology express their gratitude to each of the ***137 researchers*** who served as reviewers in 2023. They dedicated their time, effort, and expertise to analyze the submitted manuscripts, ensuring that the best works were published in the journal.

***Reviewers***

Abner Mácola

Adriano Ammirati

Amanda Carolina Zanuto

Amanda Reis

Ana Carolina Nakamura Tome

Ana Elizabeth Figueiredo

Ana Moura

André Balbi

Andre Pereira

Andreia Watanabe

Antônio Alberto Lopes

Arnauld Kaufman

Bianca Massignan

Caio Pellizzari

Camila Peña

Carla Maria Avesani

Carlos Mandarim-de-Lacerda

Carmen Tzanno

Cassiane Dezoti

Cassiano Silva

Chris Karohl

Christophe Soulage

Cibele Rodrigues

Claudia Oliveira

Claudio Gil Soares de Araujo

Conrado Gomes

Cristian Riella

Cristiane dos Santos Dias

Cristianne Alexandre

Daltro Zunino

Daniel Marchi

Dayana Bitencourt

Denise Mafra

Diêgo Fernando Figueiredo Santos

Domingos Otávio D’Avila

Eduardo Duque

Eduardo Hatanaka

Eduardo Rosa

Elieser Watanabe

Elisane Driwoski

Elizabeth Daher

Elizete Keitel

Emilia Soeiro

Erika Rangel

Eugenie R. Lumbers

Fabiana Bonato

Fabiana Hernandes

Fabiana Nerbass

Fernanda Gorayeb-Polacchini

Fernando Sales

Fernando Almeida

Fernando Antonio Colugnati

Fernando Colugnati

Fernando Henrique de Souza

Flávio Farias Filho

Geraldo Silva Junior

Gianna Kirsztajn

Giovani Gadonski

Gisele Meinerz

Hayder Fawzi

Ivan Landires

João Luiz Costa

Jocemir Ronaldo Lugon

José Abrão Cardeal Costa

José Alberto Pedroso

Jose Carolino Divino Filho

Jose Mauro Vieira Jr

José Oliveira Villar Neto

José Suassuna

José Tibúrcio Monte Neto

Juliana Abrão

Juliana Mansur

Krissia Wallbach

L. F. Quintana

Leandro Lucca

Leonardo Garcia

Leopoldo Pires

Lucas Gobetti Luz

Luciane Deboni

Luciano Drager

Lucimary Sylvestre

Lúcio Roberto Requião-Moura

Luis Eduardo Magalhaes

Luis Felipe Ferreira-Divino

Luis Gustavo Andrade

Luis Yu

Luiz Antonio Del Ciampo Jr

Marcela Sorelli

Marcelo Lopes

Marcelo Morales

Marcelo Nascimento

Marcia Franco

Marcos de Sousa

Marcus Vinicius Padua Netto

Maria Goretti Penido

Maria Helena Vaisbich

Maria Luiza do Val

Marilda Mazzali

Marion Schneider

Maryanne Silva

Mauricio Carvalho

Maycon Reboredo

Miguel Ernandes Neto

Monique Ohe

Murilo Guedes

Naila Camila Rocha

Nélia Antunes

Nelson Sass

Nilzete Bresolin

Paulo Batista

Paulo Gregório

Paulo Nogueira

Precil Neves

Rachel Armani

Rejane Bernardes

Renata Mendes

Renato Foresto

Rene Scalet dos Santos Neto

Ricardo Sesso

Roberto Manfro

Rodrigo Ramalho

Rosilene Elias

Rui Barros

Sérgio Bucharles

Silvia Botelho

Silvia Ribeiro

Thyago Moraes

Tricya Bueloni

Vandrea de Souza

Vanessa Silva

Vera Belangero

Vera Hermina Koch

Viktoria Woronik

Vinicius Delfino

Viviane Calice-Silva

Weerapong Phumratanaprapin

Welder Zamoner

